# Association of Intrinsic Brain Architecture With Changes in Attentional and Mood Symptoms During Development

**DOI:** 10.1001/jamapsychiatry.2019.4208

**Published:** 2019-12-26

**Authors:** Susan Whitfield-Gabrieli, Carter Wendelken, Alfonso Nieto-Castañón, Stephen Kent Bailey, Sheeba Arnold Anteraper, Yoon Ji Lee, Xiao-qian Chai, Dina R. Hirshfeld-Becker, Joseph Biederman, Laurie E. Cutting, Silvia A. Bunge

**Affiliations:** 1 Helen Wills Neuroscience Institute & Department of Psychology, University of California at Berkeley, Berkeley; 2 Department of Psychology, Northeastern University and McGovern Institute for Brain Research, Boston, Massachusetts; 3 Massachusetts Institute of Technology, Cambridge; 4 Vicarious FPC Inc, Union City, California; 5 Peabody College of Education and Human Development, Vanderbilt University, Nashville, Tennessee; 6 Department of Psychology, McGill University, Montreal, Quebec, Canada; 7 Massachusetts General Hospital, Boston,; 8 Harvard Medical School, Boston, Massachusetts

## Abstract

**Importance:**

Understanding the neurodevelopmental trajectory of psychiatric symptoms is important for improving early identification, intervention, and prevention of mental disorders.

**Objective:**

To test whether the strength of the coupling of activation between specific brain regions, as measured by resting-state functional magnetic resonance imaging (fMRI), predicted individual children’s developmental trajectories in terms of attentional problems characteristic of attention-deficit/hyperactivity disorder and internalizing problems characteristics of major depressive disorder (MDD).

**Design, Setting, and Participants:**

A community cohort of 94 children was recruited from Vanderbilt University between 2010 and 2013. They were followed up longitudinally for 4 years and the data were analyzed from 2016 to 2019. Based on preregistered hypotheses and an analytic plan, we examined whether specific brain connectivity patterns would be associated with longitudinal changes in scores on the Child Behavior Checklist (CBCL), a parental-report assessment used to screen for emotional, behavioral, and social problems and to predict psychiatric illnesses.^1,2^

**Main Outcomes and Measures:**

We used the strength of resting-state fMRI connectivity at age 7 years to predict subsequent changes in CBCL measures 4 years later and investigated the mechanisms of change by associating brain connectivity changes with changes in the CBCL.

**Results:**

We analyzed data from a longitudinal brain development study involving children assessed at age 7 years (n = 94; 41 girls [43.6%]) and 11 years (n = 54; 32 girls [59.3%]). As predicted, less positive coupling at age 7 years between the medial prefrontal cortex and dorsolateral prefrontal cortex (DLPFC) was associated with a decrease in attentional symptoms by age 11 years (*t*_49_ = 2.38; *P* = .01; β = 0.32). By contrast, a less positive coupling between a region implicated in mood, the subgenual anterior cingulate cortex (sgACC), and DLPFC at age 7 years was associated with an increase in internalizing (eg, anxiety/depression) behaviors by age 11 years (*t*_49_ = −2.4; *P* = .01; β = −0.30). Logistic regression analyses revealed that sgACC-DLPFC connectivity was a more accurate predictor than baseline CBCL measures for progression to a subclinical score on internalization (*t*_50_ = −2.61; *P* = .01; β = −0.29). We then replicated and extended the sgACC-DLPFC result in an independent sample of children with (n = 25) or without (n = 18) familial risk for MDD.

**Conclusions and Relevance:**

These resting-state fMRI metrics are promising biomarkers for the early identification of children at risk of developing MDD or attention-deficit/hyperactivity disorder.

## Key Points

**Question:**

Can brain imaging predict future psychiatric symptoms in children?

**Findings:**

In this 4-year longitudinal cohort study, distinct patterns of resting-state functional connectivity in healthy children predicted changes in psychiatric symptoms. Weaker positive dorsolateral prefrontal connectivity with medial prefrontal cortex predicted a better developmental trajectory for attentional symptoms, whereas weaker positive dorsolateral prefrontal connectivity with subgenual anterior cingulate cortex predicted a worse trajectory for internalizing symptoms (eg, anxiety/depression).

**Meaning:**

Brain imaging measures can contribute to early identification of children at risk for common psychiatric disorders and thus identify children in need of preventive treatments.

## Teaser

This cohort study examines the ability of resting-state networks to predict the developmental trajectory of symptoms associated with attention-deficit/hyperactivity disorder and major depressive disorder in US children.

## Introduction

The regulation of cognition and emotion is thought to depend on the top-down modulation of multiple neural circuits by the prefrontal cortex and, in particular, the dorsolateral prefrontal cortex (DLPFC).^3,4,5,6,7^ Prefrontal-dependent cognitive control mechanisms that regulate attention and mood likely play a key role in mental health.^7,8^ There is ample evidence of attenuation or failure of top-down control mechanisms in adults with depression,^9,10,11^ anxiety,^12^ and attention-deficit/hyperactivity disorder (ADHD).^13^ Given that these prevalent mental health problems often emerge during childhood and adolescence,^14,15,16^ it is important to know whether dysregulated top-down control can be detected even before behavioral symptoms are evident.

The strength of coupling between regions involved in top-down control and their targets can be measured with resting-state functional magnetic resonance imaging (rs-fMRI). Brain regions that are highly temporally correlated during rest-form resting-state networks (RSNs), which are intrinsic, spontaneous, low-frequency fluctuations in the fMRI blood oxygen level–dependent signal that define specific networks of the brain in the absence of any task.^17^ They reveal great heterogeneity in the functional organization of the brain. In fact, they may be considered “fingerprints” of the human brain, as they can accurately identify an individual from a large group (N = 126).^18^ Furthermore, RSN profiles are known to be robust and reliable.^18,19,20,21,22,23,24^

Resting-state networks are particularly relevant for studying psychiatric and pediatric populations because they are (1) task-independent, so individual differences in task performance cannot explain differences observed in the blood oxygen level–dependent data, (2) easy and fast to acquire, which make them accessible to many people, including young children and various clinical populations, and (3) and plastic, been shown to change during typical development,^25^ and can be modulated by behavioral^26,27^ or pharmacological interventions.^28,29^

An RSN that is particularly relevant for mental health is the Central Executive Network (CEN), of which the DLPFC is a key node. The CEN has been associated with externally focused attention^30^ and goal-directed behavior.^31,32,33^ In neurotypical adults, the CEN is negatively correlated (ie, anticorrelated) with the default mode network (DMN), an RSN associated with internal mentation and self-referential processing, whose key nodes include the medial prefrontal cortex (MPFC).^34,35,36,37,38^

The decoupling of these RSNs has been found to be adaptive: stronger MPFC-DLPFC anticorrelations are associated with superior cognitive control and cognitive performance in adults, such as greater working memory capacity.^39,40,41,42^ In addition, there is an increase with age in the magnitude of anticorrelations between the MPFC and DLPFC in typically developing children,^25^ which is consistent with the findings that top-down control mechanisms improve markedly over childhood and adolescence.^43^ Resting-state fMRI studies have also shown an association between diminished MPFC-DLPFC anticorrelations and cognitive impairment in ADHD.^44^

The CEN also plays a role in regulating mood through its interactions with the subgenual anterior cingulate cortex (sgACC). The sgACC is part of the affective network, which is involved in emotion processing^45,46,47,48,49,50,51,52^ and has anatomical connections to the hypothalamus, amygdala, entorhinal cortex, nucleus accumbens, and other limbic structures.^49^ There are several lines of evidence showing that top-down modulation of the sgACC is dysregulated in adults with major depressive disorder (MDD). Neuroimaging studies have reported decreased metabolisms and decreased gray matter volumes^53^ and a decreased number of glia in sgACC^54^ in patients with MDD. Furthermore, deep brain stimulation of the sgACC results in an attenuation of hyperactivation in sgACC and increased activation in previously underactive DLPFC in adults with MDD.^55^ In addition, the left DLPFC region that shows maximal anticorrelation with the sgACC in rs-fMRI has been identified as an optimal target for transcranial magnetic stimulation (TMS) of MDD.^56^ The sgACC has also been shown to exhibit decreased connectivity with cognitive control regions in children with a history of preschool depression.^57^ Finally, left DLPFC and sgACC exhibit anticorrelation in children at familial risk for MDD.^58^

In sum, prior research on patient and familial high-risk populations reveals that atypically strong functional connectivity between DLPFC and MPFC is characteristic of ADHD, whereas atypically weak connectivity between DLPFC and the sgACC is a characteristic of MDD. Here, we build on this prior work by asking whether the strength of the connectivity between these regions can predict a progression toward attentional or mood disorders in a longitudinal study of a community pediatric sample not selected for risk of ADHD or MDD.

Specifically, we tested whether DLPFC-MPFC and DLPFC-sgACC connectivity at age 7 years predict scores at age 11 years on a questionnaire used to screen children for behavioral problems, the Child Behavior Checklist (CBCL). The goals of this research were 2-fold: first, to better understand how changes in brain connectivity over childhood are associated with cognitive and affective development, and second, to evaluate the predictive validity of DLPFC-MPFC and DLPFC-sgACC connectivity for future mental health problems in children who have not been identified previously as being at elevated risk for developing a psychiatric disorder.

Numerous studies have demonstrated high reliability between the CBCL scales and actual psychiatric diagnosis.^59,60^ For example, CBCL attention problem scores are used for the screening and prediction of ADHD.^61,62^ A subthreshold elevation on the anxiety/depression subscale of the CBCL in preadolescence predicts future development of MDD.^63^ However, in conjunction with behavioral measures, neuroimaging measures may identify children at the greatest risk for developing psychiatric disorders with greater confidence and at an earlier age.

Therefore, in this study, we investigated whether rs-fMRI data could predict future CBCL scores in a community sample of 54 children. Specifically, we tested whether the individual differences in MPFC-DLPFC connectivity at age 7 years predict subsequent changes in attention 4 years later, as measured by the CBCL attentional problems measure at age 11 years. Additionally, we performed an exploratory analysis to investigate whether individual differences in sgACC-DLPFC connectivity at age 7 years predict subsequent changes in anxiety/depression 4 years later, as measured by the CBCL “internalization” and anxiety/depressed subscale at age 11 years. We preregistered our hypotheses through the Open Science Framework (OSF; https://osf.io/6cgbs/). Because of space limitations, we report only major results here; the Supplement reports additional findings, as well as a null result based on the preregistered hypotheses.

## Methods

### Participants

Ninety-four participants were included who were enrolled in a developmental longitudinal study, “Predicting Late-Emerging Reading Disability” (Vanderbilt University; principal investigator, L.C.). In this sample, 77 children (82%) met behavioral criteria for typical development; 17 children (18%) were identified as being at risk for a late-emerging reading disability. Time 1 (or baseline) data were collected from participants at age 7 years (n = 94; 41 girls [43.6]) and subsequently at 1-year intervals for 4 years. Data at time 4 were available for 54 of the original participants (57.4%) (see eMethods in the Supplement for exclusion criteria). The CBCL subscale scores at baseline did not differ significantly between those who did and did not complete the study (attentional problems, *t*_91_ = 1.0; *P* = .33; internalization, *t*_91_ = 0.51; *P* = .61; anxiety/depression, *t*_91_ = 0.41; *P* = .68; Table). The study was approved by the institutional review board at Vanderbilt University and written informed consent was received from all participants.

**Table.** Child Behavior Checklist Measures for Time 1 at Age 7 Years and Time 4 at Age 11 Years^a^

Time	Attention	Internalization	Anxiety/Depression	Withdrawn	Somatic
Time 1					
Mean (SD)	56.29 (8.13)	160.02 (13.02)	53.27 (5.3)	53.59 (5.48)	53.37 (5.48)
Subclinical (>60), No. (%)	24 (25)	10 (11)	13 (14)	14 (15)	9 (10)
Time 4					
Mean (SD)	54 (7.46)	160.13 (14.02)	53.11 (5.54)	53.15 (6.32)	53.87 (4.86)
Subclinical (>60), No. (%)	10 (20)	6 (11)	9 (17)	8 (15)	9 (17)
*P* (time 1/time 4)	0.09	0.9	0.87	0.88	0.89
Mean Change	−1.4	1.27	0.5	−0.17	1.17
Range Change	[−16 to 12] = 28	[−41 to 32] = 73	[−17 to 12] = 29	[−12 to 15] = 27	[−17 to 12] = 29

^a^ Higher scores indicate worse problems. A Child Behavior Checklist score of 60 to 70 (>1 SD, <2 SD) is generally considered to represent a medium level of symptoms (subclinical or subthreshold).

### CBCL Scoring and Data Acquisition

The CBCL assesses behavioral problems and competencies of children ages 6 to 18 years based on parental reports (eMethods in the Supplement). Data were acquired at Vanderbilt University Institute of Imaging Science on a 3-T Philips Achieva magnetic resonance spectroscopy scanner with a 32-channel head coil. One 5.9-minute resting-state echoplanar imaging scan was collected with the following parameters: TR = 2200 milliseconds, TE = 30 milliseconds, 35 slices, 3-mm isotropic voxels.

### rs-fMRI Analyses

The rs-fMRI data were analyzed in CONN (NeuroImaging Tools & Resources Collaboratory),^64^ which incorporates methods to minimize the association of head motion artifacts and allow for valid identification of correlated and anticorrelated networks^22^ (see eMethods in the Supplement for a complete description of image preprocessing/denoising, seed selection, bivariate correlation, and independent component analysis).

#### Longitudinal Analyses

Fisher-transformed r-maps from the MPFC seed were submitted to a second-level analysis of covariance regressing the changes in the CBCL measures (time 4–time 1) onto brain responses, controlling for the effect of initial severity (baseline CBCL). To create a robust prediction model that could be generalized to new cases, we performed leave-1-out cross-validation, which minimizes potential biases due to voxel selection in our predictive models (eMethods in the Supplement).

We previously found that the magnitude of MPFC-DLPFC anticorrelations grow during typical development,^25^ as does executive function, so we first implemented a whole-brain MPFC seed-based approach to determine whether MPFC-DLPFC correlations at time 1 were associated with future change in CBCL attentional symptoms after controlling for the baseline attentional score. Second, we implemented a more targeted approach by testing whether the baseline connectivity between the MPFC and the DLPFC mask derived from the previous study^25^ predicts future attentional symptoms. Finally, we ran an exploratory analysis using an independent component analysis–defined DLPFC-sgACC component to test whether the connectivity of this component predicted change of internalization symptoms and subsequently examined the internalization subscales separately: (1) anxiety/depression, (2) withdrawn behavior, and (3) somatic complaints.

#### Logistic Regression for CBCL Internalization (and Anxiety/Depression Subscale)

As per CBCL diagnostic category definitions, we subdivided participants into a “subclinical” category for individuals with a CBCL internalization (and anxiety/depression) score of 55 or greater and a “typical” category for those whose scores on this subscale fell below this cutoff based on the literature.^63^ We used a logistic regression of initial severity (baseline CBCL scores) and baseline resting-state measures combined with leave-1-out cross-validation. We did not have enough participants with subclinical scores for the CBCL attentional problems at time 4 to perform this logistic regression for that CBCL scale. Finally, we correlated the changes in connectivity with changes in CBCL measures over 4 years (from age 7 years to age 11 years).

#### Conceptual Replication/Clinical Extension

We tested the prediction model on an independent sample of 25 youths between ages 8 to 14 years identified as being at familial risk for MDD as well as 18 age-matched children without familial risk for MDD. We used baseline sgACC-DLPFC connectivity to predict the progression of CBCL anxiety/depression 3 years later (eTable in the Supplement).

## Results

### Behavioral Results and Head Motion

Between ages 7 and 11 years, 14 children (26%) had significant changes in internalizing scores (9 [17%] showing more internalizing problems at age 11 years and 5 [9%] showing fewer) and 8 children (15%) had significant changes in attentional problem scores (1 [2%] showing more attentional problems at age 11 years and 17 [3%] showing fewer). The mean (SD) number of outliers across all points was 17 of 160 (21) points. Excluding these points preserved enough data to achieve a stable estimate of RSNs^65^ (eMethods in the Supplement).

### Neuroimaging Results

Cross-sectional analyses at time 1 (N = 94) revealed that, on average, children age 7 years did not exhibit the significant negative MPFC-DLPFC correlations that are evident in adults but rather exhibited positive MPFC-DLPFC correlations on the whole, consistent with prior findings from children ages 8 to 12-years^25^ (eFigure 1 in the Supplement). We had predicted in our preregistration that there would be insufficient variance in the CBCL attentional scores to establish a significant brain-CBCL association in this sample. Indeed, we did not observe any significant correlations between the MPFC-DLPFC connectivity and CBCL attentional scores at a height threshold of *P* < .001 (*t*_92_ > 3.40) uncorrected (or even at a liberal threshold of *P* < . 01 uncorrected).

**Figure 1.**
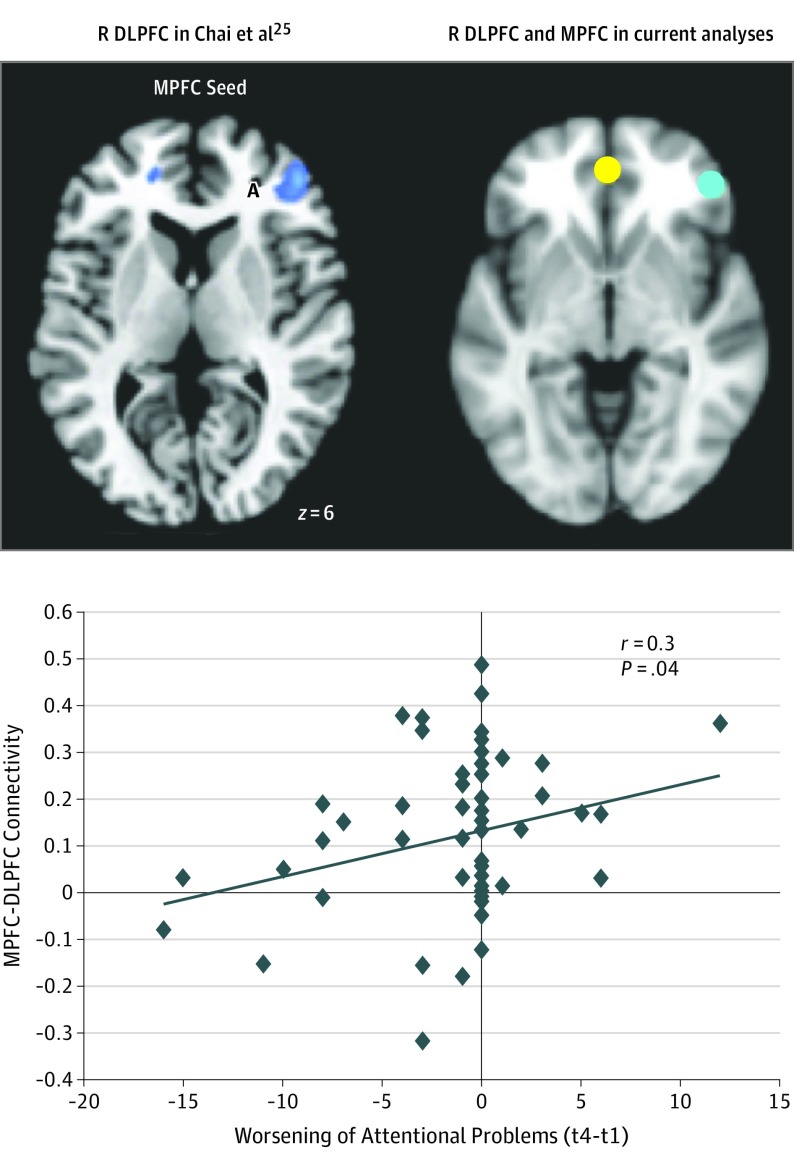
Longitudinal Prediction of Change in Attentional Problems From Ages 7 to 11 Years Baseline medial prefrontal cortex and dorsolateral prefrontal cortex (MPFC-DLPFC) (a priori mask) anticorrelations were associated with changes in attentional problems 4 years later. Negative change scores indicate improvement and positive change scores indicate decline over 4 years. The peak MPFC coordinates are −1, 47, −4 and the peak coordinates for the DLPFC mask are 46, 46, 6. R indicates right; time 1, t 1; time 4, t 4.

Although there was minimal average change in CBCL scores, there was considerable interparticipant variability in the change of CBCL scores across 4 years. Here, we used baseline neuroimaging data at age 7 years to predict CBCL change over 4 years. Less positive MPFC-DLFPC correlations at time 1 were associated with improvement of attentional problems 4 years later (*t*_49_ = 2.38, *P* = .01, controlling for medication; *t*_49_ = 1.02, *P* = .03, controlling for those children who received a diagnosis of ADHD; *t*_50_ = 2.36, *P* = .01 without controlling for participants with ADHD participants; reported *P* values are 1-sided because of our a priori and preregistered hypotheses) (eFigure 2 in the Supplement). Because we implemented this analysis using leave-1-out cross-validation, this is a prediction as opposed to a simple correlation, a distinction that is frequently lost in the neuroimaging literature.^66^ Furthermore, we found that less positive MPFC-DLPFC (a priori mask^25^) correlations at time 1 were associated with an improvement of attentional problems 4 years later (*r* = 0.3; *P* = .04; Figure 1^25^).

**Figure 2.**
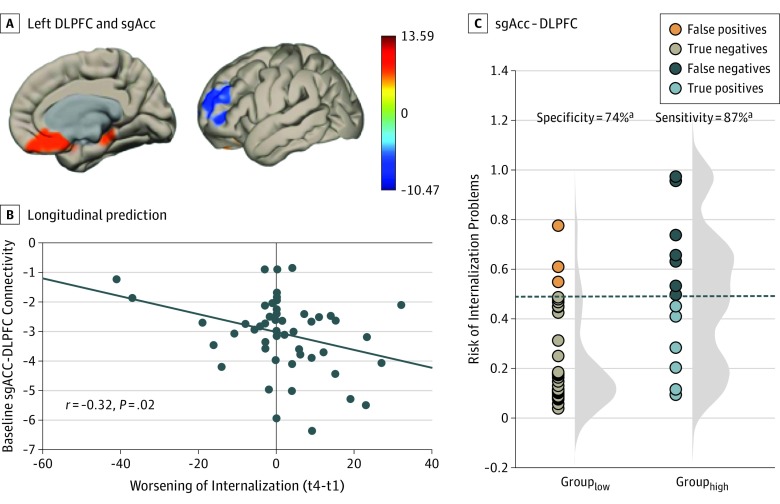
Longitudinal Prediction of Change in Internalization Problems From Ages 7 to 11 Years A, Left dorsolateral prefrontal cortex (DLPFC) and subgenual anterior cingulate cortex (sgACC) predicted change in internalization (and anxiety/depression and withdrawn subscales) such that a greater anticorrelation at time 1 (age 7 years) predicted a worsening of internalization 4 years later (age 11 years). B, Scatterplot of longitudinal prediction. Negative change scores indicate improvement and positive change scores indicate decline over 4 years. C, Logistic regression using sgACC-DLPFC to predict internalization problems at time 4 (≥55), controlling for internalization scores at time 1 (t 1). The histograms represent the distribution of the risk of internalization problems at time 4 (t 4) (as predicted by sgACC-DLPFC connectivity at time 1) displayed separately for those participants with low (left) vs high (right) internalization problem scores at time 4.

Weaker left DLPFC-sgACC connectivity at baseline predicted a greater worsening of internalization CBCL scores by time 4 (*t*_49_ = −2.4; *P* = .01; controlling for medication; Figure 2) and (*t*_49_ = −2.15; *P* = .02, controlling for ADHD) and (*t*_50_ = −2.61; *P* = .01, not controlling for ADHD or medication). Specifically, weaker left DLPFC-sgACC connectivity (or stronger anticorrelations) predicted greater worsening on the internalization subscales of anxiety/depression (*t*_49_ = −2.64; *P* = .005, controlling for medication) and withdrawn (*t*_49_ = −2.38; *P* = .01, controlling for medication). By contrast, left DLPFC-sgACC connectivity was not associated with changes in somatic complaints (*t*_49_ = −0.88; *P* = .19, controlling for medication). Based on our previous work,^58^ we had hypothesized that the sgACC-DMN connectivity would predict a worsening of internalization in our preregistration; however, this analysis did not reach current statistical threshold standards (eMethods in the Supplement).

#### Logistic Regression

Logistic regression analyses revealed that sgACC-DLPFC connectivity was a more accurate predictor than baseline CBCL measures for progression to a subclinical score on internalization (*t*_50_ = −2.61; *P* = .01; Figure 2). This analysis yielded 77% accuracy, 87% sensitivity, and 74% specificity.

#### Association of Brain Connectivity Changes With Changes in the CBCL and Conceptual Replication/Clinical Extension

**Figure 3.**
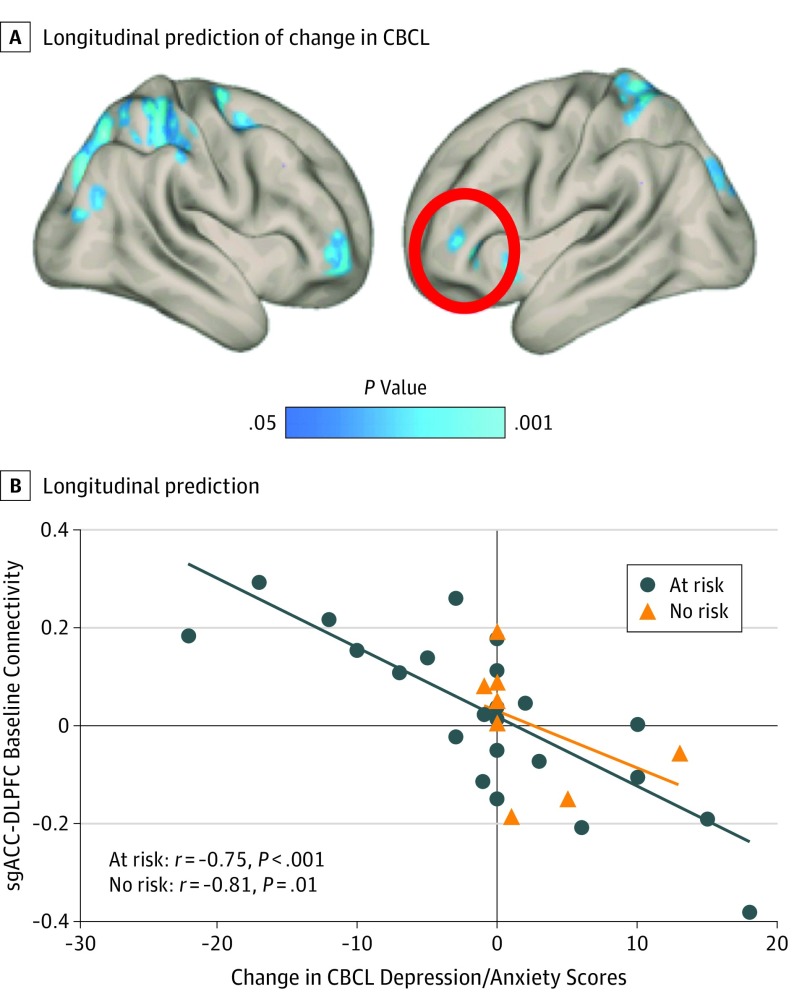
Conceptual Replication/Clinical Extension A, Longitudinal prediction of change in Child Behavior Checklist (CBCL) anxiety/depression symptoms over 3 years in children with (blue) or without (red) familial risk for depression. Baseline resting-state subgenual anterior cingulate cortex–dorsolateral prefrontal cortex (sgACC-DLPFC) connectivity predicted a change of anxiety/depression, such that less positive correlations at time 1 predicted a worsening of anxiety/depression scores 3 years later. B, Scatterplot of longitudinal prediction. Negative change scores indicate improvement, and positive change scores indicate decline over 3 years.

An increase in MPFC-DLPFC anticorrelations correlated with an improvement of CBCL attentional scores. and an increase in sgACC-DLPFC anticorrelations correlated with a worsening of CBCL anxiety/depression scores over 4 years (eFigures 3 and 4 in the Supplement). Weaker DLPFC-sgACC connectivity (or stronger anticorrelations) at baseline predicted worsening on the internalization subscale of anxiety/depression 3 years later for children at familial risk for MDD as well as a new sample of typically developing children (at-risk: *r* = −0.75; *P* < .001; controls: *r* = −0.81; *P* = .01; Figure 3 and eFigure 3 in the Supplement).

## Discussion

The RSNs at age 7 years in a community sample of children predicted the developmental trajectory of symptoms associated with ADHD and MDD at age 11 years. The variations in functional connectivity occurred in neural systems that are known to be salient for attention or mood. Weaker positive MPFC-DLPFC correlations at age 7 years predicted improved attention scores at age 11 years, whereas weaker positive sgACC-left DLPFC correlations at age 7 years predicted a worsening of MDD symptoms (internalization) at age 11 years. It is noteworthy that most children with attentional problems at age 7 years exhibited reduced symptoms at age 11 years, whereas most children with internalizing symptoms at age 7 years exhibited more symptoms at age 11 years. Thus, our functional connectivity measures appear to be sensitive to resilience and vulnerability.

The associations between specific neural networks and specific longitudinal declines are consistent with prior findings. That a stronger positive MPFC-DLPFC coupling was associated with a worse developmental trajectory for attention is consistent with the hypothesis that anticorrelated MPFC-DLPFC activation is associated with the ability to selectively focus attention on internal thoughts vs external stimuli.^67^ Weaker anticorrelations between MPFC and DLPFC, which are core nodes of DMN and CEN, respectively, may reflect an attenuation of top-down control mechanisms and an inability to allocate resources away from internal thoughts and feelings and toward external stimuli to adaptively perform difficult tasks.^67,68,69^ Thus, children age 7 years who exhibit MPFC-DLPFC anticorrelations may have the capacity to toggle between internal and external foci of attention more readily than those who do not. The failure to decouple these networks may be an early indicator of attentional problems or may preclude the development of age-appropriate attentional skills.

That stronger sgACC-left DLPFC anticorrelations predicted a future worsening of internalization, characteristic of MDD, is consistent with the MDD and at-risk literature.^70^ One study found a reduction of left DLPFC-sgACC rs-fMRI connectivity in children at familial risk for MDD, for which the at-risk group had significant anticorrelations while the not-at-risk group had positive correlations.^58^ Furthermore, left DLPFC-sgACC anticorrelations have been used to identify individually specific targets for TMS in patients with MDD.^56^ Stronger sgACC-left DLPFC anticorrelations at this young age may already reflect an attenuation or failure of top-down control mechanisms that are evident in adult MDD. Thus, the functional connectivity of specific neural systems in middle childhood forecasts individuals’ vulnerability or resilience in cognition and emotion over the ensuing 4 years of development.

These findings extend the use of neuroimaging to identify childhood neuromarkers of risk for psychopathology from highly selected children, such as those with identified familial risk, to a sample of children more representative of the population as a whole. Although children with parents who have had depression are at an elevated risk for developing depression, most children who develop depression do not come from families with an identified history of depression.^63^ Further, the longitudinal nature of this study supports the validity of using RSNs to predict the worsening of psychiatric symptoms in childhood.

Although variation in RSNs forecasts the developmental trajectory of attentional and emotional symptoms, there is strong evidence that such networks are plastic, and thus may be altered by supportive interventions. Resting-state functional connectivity is thought to reflect habitual network activations that can be remodeled by various long-term^71^ and even brief behavioral interventions^26,27,72,73^ and pharmacological interventions.^29^

### Limitations

First, although some children developed subclinical scores on CBCL measures by age 11 years, we do not know which children have subsequently converted to psychiatric diagnoses. However, elevated scores on the CBCL measures, such as internalization (including anxiety/depression), are highly predictive of near-term conversion to psychiatric diagnoses.^63^ Second, the current sample size was too small to make any meaningful interpretations for the subset of participants who moved between clinical categories over time. Third, our targeted, hypothesized-driven approach could be viewed by some readers as a limitation of the study.

## Conclusions

These findings not only further our understanding of the neurobiological vulnerabilities that foster the deterioration of mental health, but also could inform early identification and preventative treatment. Identification of risk at the level of individual children may be strengthened by large multilevel data sets that integrate multimodal neuroimaging, genetics, and social factors with new statistical tools.^74^ These findings illustrate the idea that the neurobiological seeds of future psychopathology are becoming visible and measurable in children.

## Notes

Supplement. eMethods. eTable. Table of cluster statistics and locations from Figure 3 eFigure 1. On average, children 7 years of age exhibit positive MPFC-DLPFC resting state connectivity eFigure 2. Longitudinal prediction of progression of attentional problems over four years (ages 7-11) eFigure 3. Increase in MPFC-DLPFC anticorrelations correlates with improvement of CBCL attentional scores over 4 years. eFigure 4. Increase sgACC-DLPFC anticorrelations correlates with a worsening of CBCL anxiety/depression scores over 4 years.
