# Supplementary material for: Association of Intrinsic Brain Architecture With Changes in Attentional and Mood Symptoms During Development
Source: JAMA Psychiatry. 2019 Dec 26;77(4):378–86. doi: 10.1001/jamapsychiatry.2019.4208 (PMC6990753; doi:10.1001/jamapsychiatry.2019.4208)
Supplement: Supplement. — eMethods. eTable. Table of cluster statistics and locations from Figure 3 eFigure 1. On average, children 7 years of age exhibit positive MPFC-DLPFC resting state connectivity eFigure 2. Longitudinal prediction of progression of attentional problems over four years (ages 7-11) eFigure 3. Increase in MPFC-DLPFC anticorrelations correlates with improvement of CBCL attentional scores over 4 years. eFigure 4. Increase sgACC-DLPFC anticorrelations correlates with a worsening of CBCL anxiety/depression scores over 4 years. [file jamapsychiatry-77-378-s001.pdf]

Supplementary Online Content

Whitfield-Gabrieli S, Wendelken C, Castañón AN, et al. Association of intrinsic brain architecture with changes in attentional and mood symptoms during development. *JAMA Psychiatry*. Published online December 26, 2019. doi:10.1001/jamapsychiatry.2019.4208

eMethods.

eTable. Table of cluster statistics and locations from Figure 3

eFigure 1. On average, children 7 years of age exhibit positive MPFC-DLPFC resting state connectivity

eFigure 2. Longitudinal prediction of progression of attentional problems over four years (ages 7-11)

eFigure 3. Increase in MPFC-DLPFC anticorrelations correlates with improvement of CBCL attentional scores over 4 years.

eFigure 4. Increase sgACC-DLPFC anticorrelations correlates with a worsening of CBCL anxiety/depression scores over 4 years.

This supplementary material has been provided by the authors to give readers additional information about their work.

© 2019 Whitfield-Gabrieli et al. *JAMA Psychiatry.*

eMethods

Exclusion Criteria: Children were eligible for the LERD study if they met the following general exclusion criteria: No uncorrected vision or hearing problems, no mental retardation (IQ<70), no limited proficiency in English, no brain injury (e.g., history of head trauma, meningitis, epilepsy, etc.), no severe psychiatric disorders (major depression, Tourette's syndrome, obsessive-compulsive disorder), and no ferromagnetic material in their body (e.g., braces). However, because the LERD study related to reading disability (RD) and there is a comorbidity of RD with ADHD, children with ADHD (as well as other mild psychiatric conditions, e.g., oppositional-defiant disorder, adjustment disorder, mild depression) were not excluded from participation. For the purposes of this paper, we excluded those children who were on medication and performed predictive analyses with and without the children who were diagnosed with ADHD.

ADHD Diagnosis: ADHD status was determined by DSM-IV criteria, which requires that symptoms be present in at least two settings. Therefore, two questionnaires were administered to each child's parent and teacher. For an ADHD diagnosis, participants had to meet the criterion of scoring above the 93rd percentile on at least one of two parent questionnaires/rating scales, and on at least one of the two teacher questionnaires/rating scales (ADHD Rating Scale-IV[1,2]). Participants classified as having ADHD also had to meet DSM-IV diagnostic criteria for ADHD based on Diagnostic Interview for Children and Adolescents-IV (DICA-IV[3]) interview (past or present) conducted with the parent and signs/symptoms must have been present before age 7 and have persisted for longer than 6 months. Children were only considered free of ADHD if they did not meet criteria on the parent and teacher questionnaires/rating scales used to diagnose ADHD and on the DICA-IV. Seven patients who completed the study were diagnosed with ADHD and four of them were on medication. We statistically controlled for all of the ADHD

© 2019 Whitfield-Gabrieli et al. *JAMA Psychiatry.*

subjects as well as for those four participants who were diagnosed with ADHD and were on medication. In addition, we also controlled for at-risk for reading difficulty as measured by the Woodcock Johnson scores.

CBCL Scoring: The CBCL records behavioral problems and competencies of children ages 6 to 18 years based on parental reports. Normed on a nationally representative sample of 1,753 youths, it includes the following eight empirically based syndrome scales: 1) Aggressive Behavior, 2) Anxious/Depressed, 3) Attention Problems, 4) Rule-Breaking Behavior, 5) Somatic Complaints, 6) Social Problems, 7) Thought Problems, and 8) Withdrawn/Depressed, as well as summary scores reflecting "Internalization" and "Externalization." Internalizing Problems sums the Anxious/Depressed, Withdrawn, and Somatic complaints scores, while Externalizing Problems combines Rule-breaking and Aggressive behavior. The standard scores are scaled so that 50 is average for the youth's age and gender, with a standard deviation of 10 points. Higher scores indicate greater problems. For each syndrome, scores can be interpreted as falling in normal, borderline, or clinical ranges of behavior. Researchers typically use T scores of 60-70 (>1SD <2SD) as medium level of symptoms (or "subthreshold" elevations), and T scores above 70 (>2SD) as syndromatic.

CBCL reliable change index: When calculating the changes of CBCL, we used a reliable change index (RCI). We assumed a reference Cronbach's alpha 0.90 for CBCL internalizing scores, and 0.86 for CBCL attentional problems[4,5,6].

Resting state fMRI Analyses: Resting state fMRI data were analyzed in *Conn* (http://www.nitrc.org/projects/conn)[7], which incorporates methods to both minimize the

© 2019 Whitfield-Gabrieli et al. *JAMA Psychiatry.*

influence of head motion artifacts and allow for valid identification of correlated and anti-correlated networks[8].

Preprocessing: Spatial preprocessing of functional volumes included slice timing correction, realignment, normalization, and smoothing (8mm FWHM Gaussian filter), using SPM12 (Wellcome Department of Imaging Neuroscience, London, UK; http://www.fil.ion.ucl.ac.uk/spm).

Denoising (e.g., Motion and Physiological Aliasing): To address potential spurious correlations in resting state networks caused by head motion, we used a procedure to identify problematic time points during the scan, using the Artifact Detection Tools (ART, http://www.nitrc.org/projects/artifact_detect) which is implemented in *Conn*. Specifically, an image was defined as an outlier image if the head displacement in x, y, or z direction was greater than 1.0 mm from the previous frame, or if the global mean intensity in the image was greater than 3 standard deviations from the mean image intensity for the entire resting scan. The temporal timeseries characterizing the estimated subject motion (3 rotation and 3 translation parameters, plus another 6 parameters representing their first-order temporal derivatives) and artifactual covariates (one covariate per artifactual time point consisting of 0's everywhere and a "1" for the artifactual time point), were used as nuisance regressors in the first level General Linear Model (GLM). The anatomical image for each participant was segmented into white matter, grey matter, and cerebrospinal fluid (CSF) masks using SPM12. To minimize partial voluming, the white matter and CSF masks were eroded by one voxel, which resulted in substantially smaller masks than the original segmentations[9]. The eroded white matter and CSF masks were then used as noise regions of interest (ROI). Signals from the white matter and CSF noise ROIs were extracted from the unsmoothed functional volumes to avoid additional risk of

© 2019 Whitfield-Gabrieli et al. *JAMA Psychiatry.*

contaminating white matter and CSF signals with grey matter signals. The BOLD timeseries within the subject-specific white matter mask (5 PCA parameters) and CSF mask (5 PCA parameters), were then used as temporal covariates and removed from the BOLD functional data using linear regression, and the resulting residual BOLD timeseries were band-pass filtered (0.01Hz < f < 0.10Hz).

Global signal regression, a widely used preprocessing method, was not used because it mathematically mandates negative correlations that prevent the interpretation of anticorrelations[10] and can contribute to spurious group differences in positive correlations[11]. Instead, the anatomical CompCor (aCompCor) method of noise reduction[12] as implemented in Conn and described above, allows for interpretation of anticorrelations and yields higher specificity and sensitivity compared with global signal regression[9].

Head Motion: The average number of outliers across all timepoints was 17 out of 160 timepoints. Excluding these timepoints preserved enough data to achieve a stable estimate of resting state networks[13]. Three subjects were dropped due to excessive head motion. Although rs-fMRI/behavior correlations have been called into question due to the fact that motion often correlates with the behavioral measure of interest[14], in this sample, Time 1 motion parameters did not correlate significantly with CBCL behavioral measures (or progression of CBCL measures (Time 4 – Time 1), p's > .15).

Seed Definitions: The default mode network seed was defined as a 10mm sphere around the peak coordinates from literature (MPFC: (-1, 47, -4)[15]). The selection of these coordinates was based on a number of papers illustrating that a) this MPFC seed region has significant anticorrelations with DLPFC, which correlates with executive function[16], b) there is a selective growth of anticorrelations between this MPFC seed and DLPFC in typically developing

© 2019 Whitfield-Gabrieli et al. *JAMA Psychiatry.*

children[17], and c) there is a significant reduction of MPFC-DLPFC anticorrelations in adult psychiatric populations with cognitive impairment, such as in ADHD[18], Bipolar Disorder[8], and Schizophrenia[19]. In order to define the sgACC seed to investigate the relationship between sgACC-DLPFC connectivity and the CBCL Internalization, we used Independent Component Analyses to define this component (see below).

Seed-to-voxel Bivariate Correlation: First-level correlation maps were produced by extracting the residual blood oxygen level–dependent (BOLD) time course from each seed and computing Pearson's correlation coefficients between that time course and the time course of all other voxels. Correlation coefficients were converted to normally distributed z-scores using the Fisher transformation to improve the validity of second-level General Linear Model analyses.

Previously, we reported that the MPFC is positively correlated with the right DLPFC in children (n=32, age 8)[17]. In the current sample, we applied the right DLPFC mask defined from the previous study to replicate that MPFC at Time 1 (n=94, age 7) similarly shows positive correlation with the *a priori* right DLPFC mask. The DLPFC mask was defined as a 10mm sphere around the peak coordinates from literature (46,46,6)[14.] Specifically, we performed a one-sample t-test of the MPFC-seed Fisher-transformed r-maps at Time 1 and then calculated the mean resting state correlations between the MPFC seed region and the *a priori* DLPFC mask[17] that was generated independently from the current sample (Figure S1).

ICA analyses:

© 2019 Whitfield-Gabrieli et al. *JAMA Psychiatry.*

Because we did not have an *a priori* ROI for the sgACC, we derived the sgACC-DLPFC component by performing ICA purely on the subjects' functional data, without any reference to behavioral or psychiatric measures. Because this analysis was independent of the CBCL scores there is no potential for the introduction of artifactual biases towards those components that could be more strongly associated with CBCL scores, so we didn't need to perform LOOCV. Group-level Independent Component Analyses (group-ICA[20]) were used to identify the emotional regulation network (ERN), including the sgACC. Group-level components were estimated using a 64-dimensions subject-level dimensionality reduction step, followed by 40-component group-level dimensionality reduction and fast-ICA with a hyperbolic tangent contrast function. The ERN was identified as the component with highest loading at the sgACC coordinates (5, 25, -10) (I9 seed[21]). ERN subject-level component-score maps were averaged across participants and thresholded using a combination of T>6 voxel-level "height" threshold and FWE-corrected p<.001 cluster-level threshold. This analysis resulted in a positive cluster including sgACC as well as bilateral amygdala and hippocampus, and two negative clusters in bilateral DLPFC areas. Average ICA subject-level component scores over the resulting DLPFC cluster was used in subsequent analyses as a measure of the negative association (anticorrelations) between the ERN and DLPFC for each subject, specifically between the sgACC and left DLPFC.

Longitudinal Analyses:

© 2019 Whitfield-Gabrieli et al. *JAMA Psychiatry.*

Importantly, there were no baseline brain differences between completers and non-completers at a liberal whole-brain threshold ($p = .01$ uncorrected for multiple comparisons) for all relevant ROIs (MPFC, DLPFC, sgACC).

Cross Validation: For each cross-validation iteration/fold, all subjects' data except one (the test subject) were used to perform an ANCOVA analysis looking for voxel-level associations between functional connectivity and CBCL changes. The resulting set of suprathreshold voxels (height level $p<0.001$) was then used as a mask to compute the average functional connectivity values for the test (out-of-fold) subject. This procedure was then repeated for every test subject. From the resulting average functional connectivity values the expected level of association between functional connectivity and changes in CBCL was computed. In addition, to aid in the interpretation of these results, a map of posterior probability values was computed by averaging the individual suprathreshold masks across all cross-validation folds. Because the community sample included children who had been identified as being at risk for reading difficulties, we tested whether including this variable would change the results. However, adding an 'at risk' control factor to the prediction analyses did not change the statistical significance (sgACC-DLPFC predicting internalizing controlling for medication: $T(49) = -2.41$, p (two-sided) = 0.020, as compared with sgACC-DLPFC predicting internalizing, controlling for medication & at risk status: $T(48) = -2.45$, p (two-sided) = 0.018; MPFC-DLPFC predicting attention, controlling for medication: $T(49) = 2.38$, p (two-sided) = 0.021, as compared with MPFC-DLPFC predicting attention, controlling for medication & at risk status: $T(48) = 2.20$, p (two-sided) = 0.032.)

© 2019 Whitfield-Gabrieli et al. *JAMA Psychiatry.*

Null Result: Based on our previous work (Chai et al. Biological Psychiatry, 2016, Figure 2), we initially hypothesized the sgACC-DMN connectivity would predict worsening of internalization in our pre-registration, however this analysis did not reach current statistical threshold standards.

Replication and Clinical Extension:

We analyzed data from 25 youth between ages 8-14 who were identified as being at familial risk for Major Depressive Disorder, as well as 18 age-matched children who were not identified as being at risk. Participants were tested at two timepoints 3 years apart. The baseline pediatric sample was defined in Chai et al., 2016[22] and the follow up resting state prediction of conversion analyses are described in Shapero et al., 2019[23]; Hirshfeld-Becker et al., 2019[24]. In order to replicate and extend our initial results, we again used sgACC seed-based prediction analysis to replicate the finding that weaker DLPFC-sgACC connectivity (or stronger anticorrelations) at baseline predicted greater worsening on the internalization subscale of anxiety/depression three years later for both children at familial risk for MDD as well as a new sample of typically developing children. We also noted other clusters within the CEN that predicted change of CBCL anxiety/depression symptoms, such bilateral parietal cortices.

© 2019 Whitfield-Gabrieli et al. *JAMA Psychiatry.*

Region	BA	Voxels	MNI (x,y,z mm)	R	p Value
Right Superior Parietal Lobule	7	130	+26 -38 +58	-.85	0.01
Left Superior Parietal Lobule	7	72	-24 -50 +64	-.80	0.02
***Dorsolateral Prefrontal Cortex DLPFC**	9	72	+44 +52 +00	-.81	0.02
Right Middle Frontal Gyrus	6	58	+30 -02 +50	-.78	0.02
Lateral Occipital Cortex **	19	8	+24 -78 +30	.12	0.43
Right Motor Cortex**	4	8	+46 -15 +39	.05	0.86

eTable. Table of cluster statistics and locations from Figure 3 which is a longitudinal prediction of progression of CBCL anxiety/depression problems over three years in children who are at familial risk for depression. Baseline resting state connectivity for the sgACC and these clusters predicted progression of anxiety/depression. The replication of note is that greater anticorrelation of baseline sgACC-DLPFC predicted worsening of anxiety/depression three years later.

© 2019 Whitfield-Gabrieli et al. *JAMA Psychiatry.*

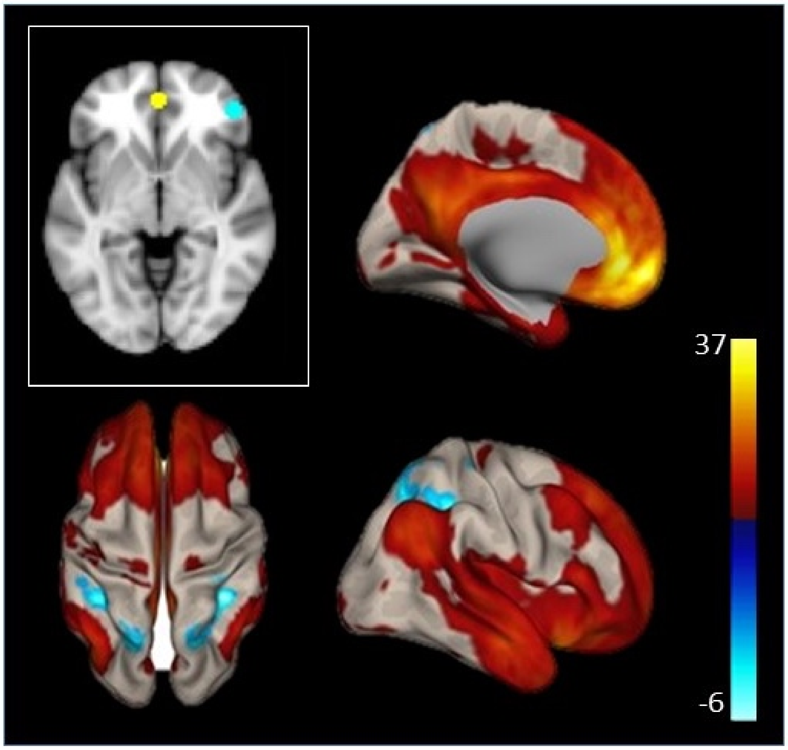

eFigure 1. On average, children 7 years of age exhibit positive MPFC-DLPFC resting state connectivity (n=94, age 7): a) MPFC seed (yellow) and DLPFC mask from previous study (blue) b) Whole brain MPFC seed driven resting state functional connectivity map. *Pre-registered hypothesis

© 2019 Whitfield-Gabrieli et al. *JAMA Psychiatry.*

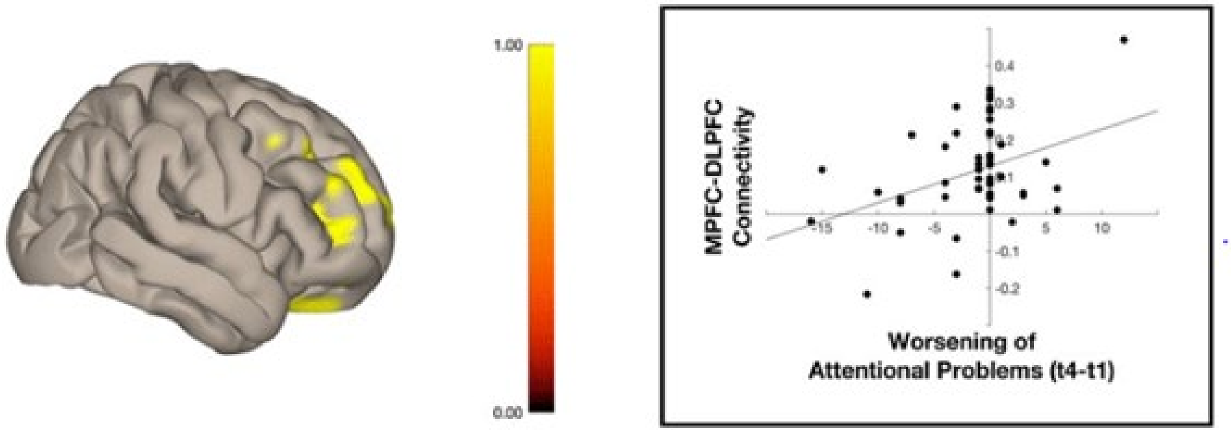

eFigure 2. Longitudinal prediction of progression of attentional problems over four years (ages 7-11). Weaker T1 MPFC-DLPFC anticorrelations predicted worsening of attentional problems 4 years later. Note: negative change scores indicate improvement, and positive change scores indicate decline over four years. *Pre-registered hypothesis

© 2019 Whitfield-Gabrieli et al. *JAMA Psychiatry.*

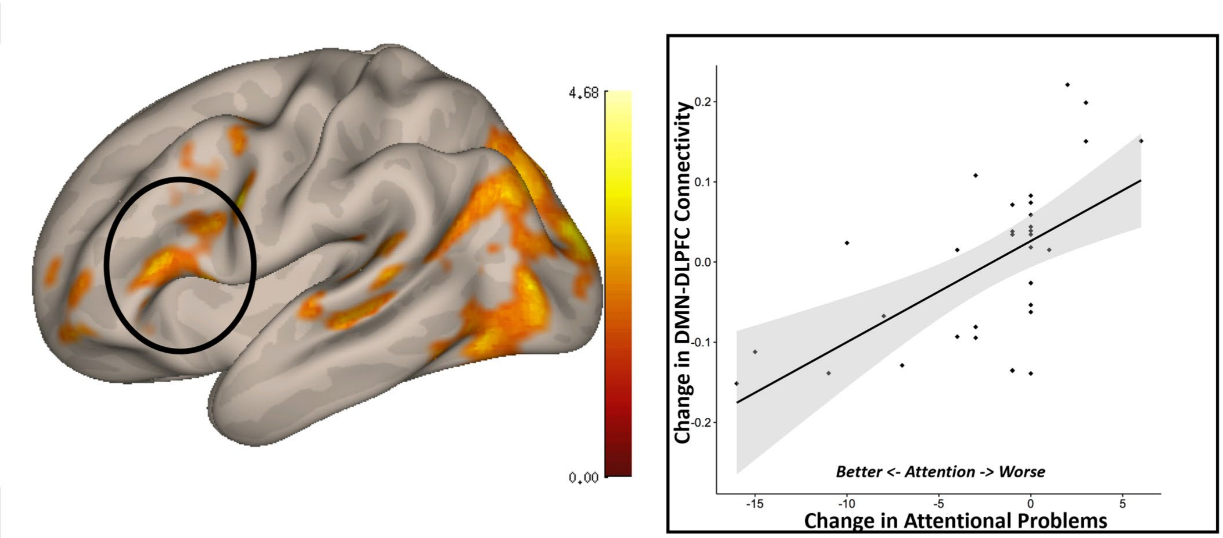

eFigure 3. Increase in MPFC-DLPFC anticorrelations correlates with improvement of CBCL attentional scores over 4 years.

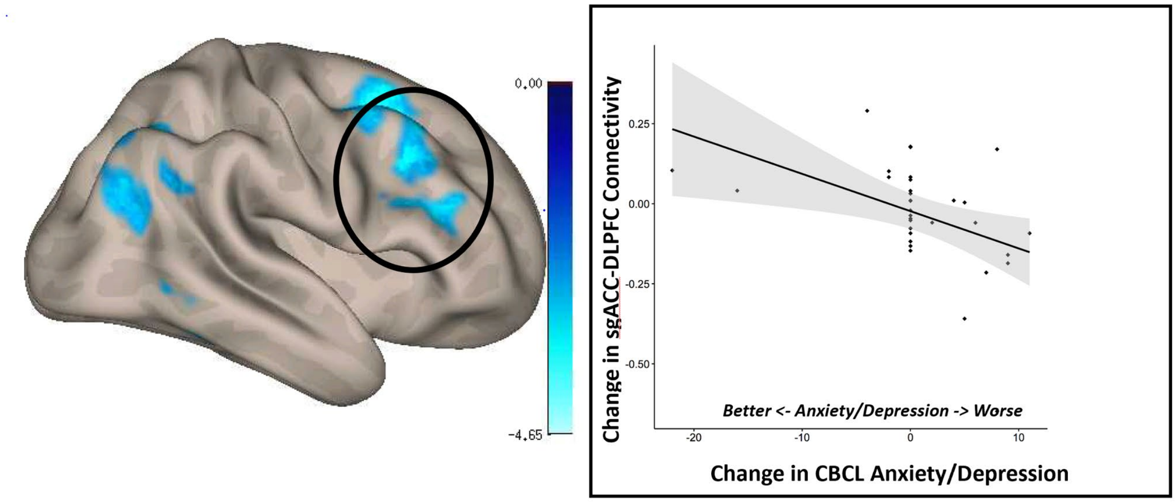

eFigure 4. Increase sgACC-DLPFC anticorrelations correlates with a worsening of CBCL anxiety/depression scores over 4 years.

© 2019 Whitfield-Gabrieli et al. *JAMA Psychiatry.*

References

1. DuPaul G., Anastopopoulos A., Power T., Reid R, Ikeda M., McGoey K. (1998) Parent Ratings of Attention-Deficit/Hyperactivity Disorder Symptoms: Factor Structure and Normative Data. Jour of Psychopathology and Behavioral Assessment 20:1
2. Conners K. Conners Third Edition (Conners 3)
3. Reich A., (2000) Journal of the American Academy of Child & Adolescent Psychiatry. 39:59-66.
4. Achenbach, T.M., & Rescorla, L. A. (2001). Manual for the ASEBA School-Age Forms and Profiles.Burlington, VT: University of Vermont, Research Center for Children, Youth, and Families. ISBN 0-938565-73-7
5. Achenbach, T. M., Dumenci, L., & Rescorla, L. A. (2003). Are American children's problems still getting worse? A 23-year comparison. Journal of Abnormal Child Psychology, 31(1), 1-11.
6. Albores-Gallo, L., Lara-Muñoz, C., Esperón-Vargas, C., Cárdenas, J. Z., Pérez, A. S., & Villanueva, G. C. (2007). Validity and reliability of the CBCL/6-18. Includes DSM scales. Actas espanolas de psiquiatria, 35(6), 393-399.
7. Whitfield-Gabrieli S, Nieto Castanon A. (2012) Conn: A functional connectivity toolbox for correlated and anticorrelated brain networks. Brain Connectivity. 2:125–141.
8. Chai XJ, Whitfield-Gabrieli S, Shinn AK, Gabrieli JD, Nieto Castanon A, McCarthy JM, et al. (2011) Abnormal medial prefrontal cortex resting-state connectivity in bipolar disorder and schizophrenia. Neuropsychopharmacology. 36:2009–2017.
9. Chai, X. J., Castanon, A. N., Ongur, D., & Whitfield-Gabrieli, S. (2012) Anticorrelations in resting state networks without global signal regression. Neuroimage, 59, 1420–1428.
10. Murphy K, Birn RM,Handwerker DA, Jones TB, Bandettini PA. (2009) The impact of global signal regression on resting state correlations: Are anti-correlated networks introduced? Neuroimage 44:893–905
11. Saad ZS, Gotts SJ, Murphy K, Chen G, Jo HJ, Martin A, Cox RW (2012): Trouble at rest: How correlation patterns and group differences become distorted after global signal regression. Brain Connect 2:25–32.
12. Behzadi, Y., Restom, K., Liau, J., & Liu, T. T. (2007). A component based noise correction method (CompCor) for BOLD and perfusion based fMRI. Neuroimage, 37, 90–101.
13. Van Dijk KRA, Hedden T, Venkataraman A, Evans KC, Lazar SW, Buckner RL (2010): Intrinsic functional connectivity as a tool forhuman connectomics: Theory, properties, and optimization. J Neurophysiol103:297–321
14. Siegel JS, Mitra A, Laumann TO, Seitzman BA, Raichle M, Corbetta M, and Snyder A (2016) Data quality influences observed links between functional connectivity and behavior. Cerebral Cortex. 2016 Aug 22. [Epub ahead of print]
15. Fox, M. D., Snyder, A. Z., Vincent, J. L., Corbetta, M., Van Essen, D. C., & Raichle, M. E. (2005). The human brain is intrinsically organized into dynamic, anticorrelated functional networks. Proceedings of the National Academy of Sciences, U.S.A., 102, 9673–9678.

© 2019 Whitfield-Gabrieli et al. *JAMA Psychiatry.*

16. Bunge, S., & Toga, A. (2013) Introduction to section II: Frontal lobe development. Principles of frontal lobe function, 93-98.
17. Chai J., Ofen N., Gabrieli J.D.E., Whitfield-Gabrieli S. (2014). Development of resting-state functional connectivity of the default network: maturation of anticorrelated networks Journal of Cognitive Neuroscience, 26: 501-513.
18. Mattfeld A., Gabrieli J., Biederman J., Spencer T, Brown A., Kotte A. , Kagan E., Whitifeld-Gabrieli S. (2014). Resting-state functional connectivity in a longitudinal study of ADHD children grown-up reflects persistent diagnostic status. Brain, 137:2423-8
19. Whitfield-Gabrieli, S., Thermenos, H., Milanovic, S., Tsuang, M., Faraone, S., McCarley, R., Shenton, M., Green, A., LaViolette, P., Wojcik, J., Gabrieli, J.D.E., Seidman, L. (2009) Hyperactivity and hyperconnectivity of the default network in schizophrenia and in first degree relatives of persons with schizophrenia, Proceedings of the National Academy of Sciences of the United States of America, 106:1279-84
20. Calhoun V., Adali T., Pearlson G., and Pekar J., (2001) A Method for Making Group Inferences From Functional MRI Data Using Independent Component Analysis Hum. Brain Map., 14:140-151
21. Kelly, A.C., Di Martino, A., Uddin, L.Q., Shehzad, Z., Gee, D.G., Reiss, P.T., Margulies, D.S., Castellanos, F.X. and Milham, M.P., (2009) Development of anterior cingulate functional connectivity from late childhood to early adulthood. Cerebral cortex, 19:640-657.
22. Chai X.J., Hirshfeld-Becker D., Doehrmann O., Leonard J., Biederman J., Gabrieli J, Whitfield-Gabrieli S. (2016) Altered Intrinsic Functional Brain Architecture in Children at Familial Risk of Major Depression. Biological Psychiatry, 80:849-858.
23. Shapiro, B.G., Chai, X.J., Vangel, M., Biederman, J., Hoover, C.S., Whitfield-Gabrieli, S., Gabrieli, G.D.G., Hirshfeld-Becker, D. (2019) Neural markers of depression risk predict the onset of depression. NeuroImage. 285:31-39.
24. Hirshfeld-Becker, D., Gabrieli, J.D., Shapero, B., Biederman, J., Whitfield-Gabrieli, S., Chai, X.J. (2019) Intrinsic Functional Brain Connectivity Predicts Onset of Major Depression Disroder in Adolescence: A Pilot Study. Brain Connect.

© 2019 Whitfield-Gabrieli et al. *JAMA Psychiatry.*
